# Visualizing complex processes using a cognitive-mapping tool to support the learning of clinical reasoning

**DOI:** 10.1186/s12909-016-0734-x

**Published:** 2016-08-22

**Authors:** Bian Wu, Minhong Wang, Tina A. Grotzer, Jun Liu, Janice M. Johnson

**Affiliations:** 1 Department of Educational Information Technology, East China Normal University, KM&EL Lab, The University of Hong Kong, Hong Kong SAR, Hong Kong; 2 KM&EL Lab, Faculty of Education, The University of Hong Kong, Hong Kong SAR, Hong Kong; 3 Harvard Graduate School of Education, Harvard School of Public Health, Boston, MA USA; 4 Department of Nephrology, Shanghai Jiao Tong University Affiliated First People’s Hospital, Shanghai, China; 5 School of Public Health, Li Ka Shing Faculty of Medicine, The University of Hong Kong, Hong Kong SAR, Hong Kong

**Keywords:** Clinical reasoning, Cognitive mapping, Computers, Technology

## Abstract

**Background:**

Practical experience with clinical cases has played an important role in supporting the learning of clinical reasoning. However, learning through practical experience involves complex processes difficult to be captured by students. This study aimed to examine the effects of a computer-based cognitive-mapping approach that helps students to externalize the reasoning process and the knowledge underlying the reasoning process when they work with clinical cases. A comparison between the cognitive-mapping approach and the verbal-text approach was made by analyzing their effects on learning outcomes.

**Methods:**

Fifty-two third-year or higher students from two medical schools participated in the study. Students in the experimental group used the computer-base cognitive-mapping approach, while the control group used the verbal-text approach, to make sense of their thinking and actions when they worked with four simulated cases over 4 weeks. For each case, students in both groups reported their reasoning process (involving data capture, hypotheses formulation, and reasoning with justifications) and the underlying knowledge (involving identified concepts and the relationships between the concepts) using the given approach.

**Results:**

The learning products (cognitive maps or verbal text) revealed that students in the cognitive-mapping group outperformed those in the verbal-text group in the reasoning process, but not in making sense of the knowledge underlying the reasoning process. No significant differences were found in a knowledge posttest between the two groups.

**Conclusions:**

The computer-based cognitive-mapping approach has shown a promising advantage over the verbal-text approach in improving students’ reasoning performance. Further studies are needed to examine the effects of the cognitive-mapping approach in improving the construction of subject-matter knowledge on the basis of practical experience.

## Background

Clinical reasoning is at the core of medical expertise. It can be defined as inferential processes for collecting and analyzing data and making judgments or decisions about the diagnosis or treatment of patient problems [1]. Internship programs, case-based sessions, problem-based learning programs have been widely employed in education programs to provide opportunities for learning clinical reasoning via working with authentic problems in real-world or simulated environments [2, 3]. However, desired learning outcomes are not easy to achieve. Problem-solving practice often involves complex, context-specific processes in search for information on multiple aspects, integration of problem information with subject-matter knowledge, and reasoning with interactive components [4]. The complexity will increase in solving problems with incomplete information. With limited abilities to communicate complex ideas and capture key aspects of practical experience, many learners have difficulties making adequate understanding of the experience and transferring useful knowledge for reuse in new situations [5]. To address the problem, the following two issues have to be taken into account.

Firstly, making sense of practical experience requires the use of language or other forms of representation for communication of complex ideas. For example, think-aloud approaches were used for experts and novices to verbalize and explain their thinking and actions when they process and integrate patient information with relevant knowledge to perform reasoning and diagnostic tasks [1, 6]. Prior studies have indicated that verbal text alone is limited in representing the understanding of complex issues, and a diagram is sometimes worth a thousand words [7]. Graphic forms, if used appropriately, can reduce the cognitive load via meaningful representation of abstract ideas. The issue of cognitive load in learning with complex problems has received increased attention [8]. Recent research has highlighted the importance of making complex thinking visible to students in order to reduce the cognitive work in teaching and learning of clinical reasoning [9].

Visual representations or graphic forms have advantages in representing complex thinking and cognition in flexible ways. By representing information both verbally and spatially, graphic forms may enable more meaningful representation and efficient cognitive processing of complex issues than text messages alone. Among various forms, concept mapping, i.e., using a schematic to represent a set of concepts as nodes and their relationships as links, has been increasingly used as a teaching and learning strategy [10]. In medical education, concept mapping has been found to enable meaningful learning and systematic thinking by allowing learners to represent their conceptual understanding in flexible formats for reflection, discussion, and assessment [11]. However, concept mapping alone is found to be inadequate in supporting complex problem-solving processes, especially for eliciting and representing the procedure of applying knowledge to practice [12].

Secondly, making sense of practical experience requires attention to key aspects of the experience that are essential for improving the problem-solving performance. A person’s problem-solving performance is found to be influenced by his/her problem-solving and reasoning skills as well as subject-matter knowledge [13]. The former concerns the hypothesis-driven reasoning process typically used by novices to solve diagnostic problems, i.e., reasoning by generating and testing hypotheses to account for the data [14]. The latter concerns not only specific concepts or principles, but also the organization of knowledge into a systematic structure for meaningful understanding and flexible application [13, 15].

Accordingly, learners wishing to improve their clinical problem-solving performance need to make sense of their experience with a focus on two essential aspects: the *reasoning process* (i.e., how the problem is solved via relevant reasoning and actions) and the *knowledge underlying the reasoning process* (i.e., how relevant knowledge is identified and organized into a systematic structure). Making sense of the reasoning process is related to performance-based assessment increasingly employed in medical education, where a set of performance measures such as pertinent findings, performed actions, and generated hypotheses are used to assess learners’ clinical reasoning abilities [16].

### A pilot study

A prior study proposed a computer-based cognitive mapping approach that helped students to externalize the complex reasoning process and the knowledge underlying the reasoning process when they worked with clinical cases [17, 18]. By allowing learners to visualize a set of key elements of cognition in a cognitive map, this approach aimed to facilitate the learning of clinical reasoning via making meaningful understanding of practical experience. The approach extended traditional concept mapping by guiding learners’ attention to key aspects of cognition involved in clinical reasoning and in construction of knowledge underling the reasoning process. It is a pilot study focusing on technical details and initial evaluation of the approach. In particular, it reported the design and implementation of the cognitive mapping tool, learners’ feedback, and pre-post improvement made by learners using the tool in a four-week period. Students claimed to make moderate learning progress through the study. They were found to make significant improvement in their learning products from the beginning to the end of the study. The positive results showed the feasibility and usefulness of the cognitive mapping approach. Nevertheless, the evidence from this pilot study is limited by a lack of a control group.

### The present study

Built on the pilot study, the present study aimed to further examine the effects of the computer-based cognitive mapping approach by comparing it to a traditional verbal-text approach. In doing so, the cognitive mapping tool developed in the pilot study was used in the present study after some refinement of the interfaces based on learners’ feedback. Most importantly, a control group design was adopted in the present study; learners in the experimental group used the cognitive-mapping approach, while those in the control group used the verbal-text approach, to elicit their thinking and actions when they performed clinical reasoning and problem-solving tasks in a simulated environment. The study aimed to answer the following research questions (RQs).

RQ1: Will learners using the cognitive-mapping approach perform better than those using the verbal-text approach in their problem-solving performance?

To answer this question, the cognitive maps built by the experimental group and the verbal text made by the control group were analyzed to examine learners’ problem-solving performance reflected in the reasoning process and the construction of knowledge underlying the reasoning process.

RQ2: Will learners using the cognitive-mapping approach perform better than those using the verbal-text approach in the subject-matter knowledge posttest?

To answer this question, learners’ test scores were analyzed and compared between the two groups.

RQ3. Will learners using the cognitive-mapping approach have more positive perceptions than those using the verbal-text approach reflected in their perceived learning gains and comments on the learning program?

To answer this question, learners’ perceptions of the learning gains and comments on the learning program collected from a survey were analyzed and compared between the two groups.

## Methods

### Participants

Fifty-two students from two medical schools participated in the study. Their participation in this study was completely voluntary. Twenty-six participants from school A were in the third year (11, 42.3 %), fourth year (9, 34.6 %), or fifth year (6, 23.1 %) of their 7-year medical school curriculum. Those from school B were in the third year (2, 7.7 %), fourth year (13, 50 %), fifth year (9, 34.6 %), or higher (2, 7.7 %) of their 7-year medical school curriculum. Students from both schools had fundamental medical knowledge and problem-based learning experience. The background knowledge of all the participants was assumed to be comparable given the similar curriculum standards and entry requirements adopted by both schools.

Considering that students from the same school might have more chance to interact during the experiment, the participants were not randomly assigned to one of the two conditions. Instead, the two schools were randomly assigned to the two environments. Students from school A were assigned to the *experimental group*, and those from school B to the *control group*. The pretest scores showed no significant difference between the two groups in their prior knowledge. The demographic data showed that there were more senior students in the control group. Senior students (fourth year or higher) in both schools had received similar clinical training at the same large urban teaching hospital.

### Learning materials

Students in both groups were asked to work with simulated cases of kidney disease in an online system. Five cases were provided, including a sample case for demonstration and pre-study practice and the other four for independent study by learners. All the cases were adapted from clinical practice and academic references, and were determined by the experts to be at a similar level of complexity.

Learners could access the information of each case via the system. The information was categorized into patient history, physical examinations, lab tests, imaging records, patient state, and prescription history. In addition to original information, learners could order clinical examinations or tests to obtain additional information of the case. For each case, learners in both groups were required to report their learning process involving five elements: data capture, hypotheses formulation, reasoning with justifications, concept identification, and concept relationships. The first three elements reflect the *reasoning process* and the latter two reflect the *construction of knowledge* underlying the reasoning process. Learners in the *experimental* group used the cognitive-mapping tool to represent the learning process in a cognitive map, while learners in the *control* group used a note-taking tool to report the learning process in verbal text.

**Fig. 1:**
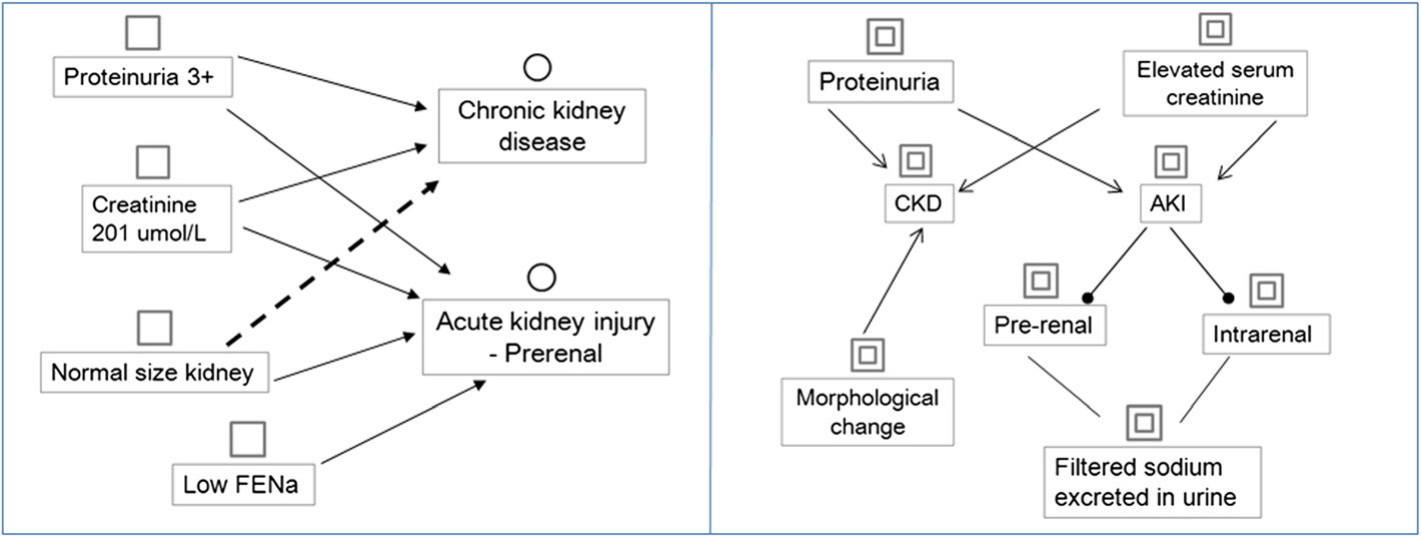
Description of data: Captured data. Generated hypothesis. Reasoning link (support). Reasoning link (against). Underlying concept. Causal relation between concepts. Hierarchical relation between concepts. Cross-link relation between concepts

A simplified example of using the cognitive-mapping approach for learning with a clinical case is shown in Fig. 1; the left part represents the reasoning process, and the right part represents the construction of knowledge underlying the reasoning process. The patient was observed to have proteinuria and increased serum creatinine. Based on the two symptoms, the learner recalled relevant knowledge about chronic kidney disease (CKD) or acute kidney injury (AKI), as represented in the right part of the map. Accordingly, two hypotheses, chronic kidney disease and acute kidney injury, were generated; the former was rejected and the latter was supported with further information about the normal size of the kidney, as outlined in the left part of the map. During the process, the learner recalled other knowledge relevant to the diseases. As shown in the map, chronic kidney disease may cause morphological changes in the kidney; acute kidney injury may cause prerenal and intrarenal diseases, and fractional excretion of sodium (FENa) can be used for differentiation.

### Procedure

At the beginning of the learning program, a questionnaire survey and a pretest were administered to collect the participants’ demographic information and assess their prior knowledge. A tutorial of how to work with clinical cases and make sense of the reasoning process and the underlying knowledge using the five-element template was offered to both groups. Next, a face-to-face demonstration of how to use the learning system and the given tool was provided to each of the two groups. After practicing with the sample case to become familiar with the learning approach and tool, students started their individual study of the other four cases.

The participants were required to complete their independent study of the four cases within 4 weeks. They were asked to pace themselves and were advised to spend about 5 h per case. For each case, students interacted with the case to capture critical information, perform clinical examinations to obtain further information, make clinical reasoning and diagnosis, and identify the knowledge underlying the reasoning process. Given incomplete information of the case, students need to go through several rounds of clinical examinations to collect sufficient data, and their selection of a clinical examination was based on their initial analysis of existing data. Moreover, students were asked to use the given tool (cognitive-mapping, note-taking) to report their learning process involving the five elements. A summative report on the analysis and resolution of the case prepared by the domain experts could be accessed by the student after he or she had completed that case. No other feedback was provided to learners in either group. During the study period, there was no teacher involvement except for assistance with technical problems. Learners were prompted by the teacher to engage actively in the learning tasks, and they were allowed to utilize other learning materials or resources external to the system.

At the end of the program, a survey was administered to collect learners’ perceived learning gains and their comments on the learning program. Furthermore, learners were required to complete a posttest to assess their subject-matter knowledge at the end of the study. In sum, students spent five weeks on the experiment, including four weeks for the independent study with four cases, and one week for pretest, posttest, and pre-study instruction and practice with the learning system.

### Measures

#### Problem-solving performance

The cognitive maps and verbal text created by learners were analyzed to assess their problem-solving performance based on a set of predefined scales and rubrics adapted from prior studies [19–23]. The rubrics consisted of five components: data capture, hypotheses formulation, reasoning for justifying or refuting hypotheses, concept identification, and concept relationships. As shown in Table 1, each component was scored on a five-level scale between 0 (lowest) and 1 (highest). The average score for data capture, hypotheses formulation, and reasoning reflected the performance in the *reasoning process*, while the average score for concept identification and concept relationships reflected the performance in the *construction of knowledge* underlying the reasoning process. The average of the scores for all five components reflected the overall performance.

**Table 1:** Rubrics for assessing problem-solving and knowledge-construction processes

Component	Description
Problem-solving process	
1) Data capture	Identify critical data from patient information 0: no critical, well-described data 1: mostly critical, well-described data
2) Hypotheses formulation	Formulate hypotheses 0: no plausible hypotheses 1: plenty of plausible, differential diagnostic hypotheses in a strategic sequence from general to more specific
3) Reasoning	Perform reasoning to support or reject hypotheses 0: unjustified, incorrect reasoning 1: sufficient, well-justified reasoning
Knowledge-construction process	
4) Concept identification	Trigger concepts from the case information 0: no or irrelevant concepts 1: plenty of closely related, problem solving–oriented concepts
5) Concept relationships	Construct relations between concepts 0: no or incorrect concept relations 1: plenty of well-organized, thought-provoking relations

#### Subject-matter knowledge test

Pretest and posttest were used to assess learners’ knowledge about kidney function and problems. The questions in both tests were adapted from relevant textbooks and comparable to those used by the medical schools. The two tests used different questions, but at the same level of difficulty. Their validity was confirmed by domain experts. Each test included three multiple-choice questions, ten extended matching questions, and four short essay questions. The scores ranged from 0 (incorrect) to 4 (full mark) for each question, with a test range of 0 to 68 rescaled to the range of 0 to 1. The essay questions were assessed based on a five-level scale including 0: little argument and evidence; 1: argument with irrelevant evidence; 2: argument supported by limited evidence; 3: argument supported by more evidence; and 4: argument supported by sufficient evidence.

The test papers and learning products of the two groups were assessed by two domain experts, both of whom were blind to student identification, test information (i.e., whether the test was pretest or posttest), and learning product information (i.e., whether the product was for the first case or the last case) of both groups. The inter-rater reliability computed using Cohen’s kappa reflected a high degree of agreement and consistency between the raters, that was 0.93 for test papers and 0.91 for learning products (both significant at the 0.01 level).

#### Learner perceptions and comments

A questionnaire survey was administered to collect learners’ self-perceived learning gains with regard to reasoning and problem-solving skills as well as construction of knowledge underlying the reasoning process, using a 5-point Likert scale ranging from 0 (no progress) to 4 (substantial progress). The measuring items were adopted from the Student Assessment of their Learning Gains (SALG) instrument [24]. Internal consistency analysis using Cronbach’s alpha confirmed that all subscales were reliable (0.85 for reasoning and problem solving, 0.79 for construction of knowledge). The survey also included two open-ended questions: 1) positive and negative comments on the learning program; and 2) suggestions for improvement of the learning program.

### Data analysis

All the participants completed the learning program. For each group, paired-sample t-tests were used to compare learners’ problem-solving performance between the first and last cases and to compare the pretest and posttest scores. Further, the differences between the two groups in the problem-solving performance, test scores, and perceived learning gains were examined using independent *t*-tests. Finally, learners’ responses to the two open-ended questions were summarized.

## Results

### Problem-solving performance

The learning products generated in both groups for the first and last cases were analyzed. Table 2 presents the descriptive statistics for the performance and comparison of means of each measure between the first and last cases based on paired-sample *t*-test results.

**Table 2:** Comparison of learning products between the first and last cases (range: 0–1; *n* = 26 for each group)

		First case		Last case		Paired-sample *t*-tests			
		M	SD	M	SD	Paired difference	t	df	p
RP	Exp	.53	.15	.67	.22	.14	2.63	25	.025^*^
	Ctrl	.38	.11	.43	.09	.05	1.31	25	.212
1) DAT	Exp	.64	.21	.82	.16	.18	3.73	25	.004^**^
	Ctrl	.55	.22	.66	.13	.11	2.12	25	.054
2) HYP	Exp	.52	.21	.59	.28	.07	.90	25	.391
	Ctrl	.30	.11	.29	.09	-.02	-.56	25	.583
3) REA	Exp	.43	.20	.59	.30	.16	1.75	25	.111
	Ctrl	.30	.11	.32	.15	.02	.37	25	.720
*CK*	Exp	.13	.17	.34	.28	.21	2.73	25	.021^*^
	Crl	.39	.14	.42	.15	.03	.23	25	.862
4) CON	Exp	.18	.23	.39	.28	.21	2.52	25	.031^*^
	Ctrl	.41	.16	.45	.20	.04	.04	25	.435
5) COR	Exp	.07	.12	.30	.29	.23	2.65	25	.024^*^
	Ctrl	.36	.16	.39	.16	.03	1.00	25	.336
Overall	Exp	.39	.13	.55	.25	.16	2.61	25	.026^*^
	Ctrl	.38	.13	.39	.13	.02	.56	25	.583

DAT: data capture; HYP: hypotheses formulation; REA: reasoning; CON: concept identification; COR: concept relationships

RP: reasoning process; CK: construction of knowledge

Exp: experimental group; Ctrl: control group

*p <0.05; **p <0.01

For the *experimental* group, learners improved from the first to the last case in several aspects involving data capture, concept identification, concept relationships, and overall performance (paired difference = 0.16, t = 2.61, df = 25, *p* < 0.05). The effect size indicated major progress in overall performance (Cohen’s d = 0.82). When categorizing the five composite measures into the reasoning process and the construction of knowledge underlying the reasoning process, improvement in both dimensions was found from the first case to the last case. For the *control* group, there was no significant difference between the first and last cases in any aspect of the problem-solving performance.

Table 3 shows the independent *t*-test results comparing the learning products for the last case between the two groups. Learners in the experimental group were found to outperform those in the control group in several aspects involving data capture, hypotheses formulation, reasoning with justifications, and overall performance (mean difference = 0.16, t = 2.89, df = 49, *p* < 0.05). The effect size indicated a major effect of the cognitive-mapping approach on overall performance (Cohen’s d = 0.82). When categorizing the five composite measures into the reasoning process and the construction of knowledge underlying the reasoning process, learners in the cognitive-mapping group were found to outperform those in the verbal-text group in the reasoning process, but not in the construction of knowledge underlying the reasoning process.

**Table 3:** Comparison of learning products for the last case between the two groups

		Learning product		Independent *t*-tests			
		M	SD	Mean difference	t	df	p
RP	Exp	.67	.22	.24	3.43	49	.005**
	Ctrl	.43	.09				
1) DAT	Exp	.82	.16	.16	2.76	50	.011*
	Ctrl	.66	.13				
2) HYP	Exp	.59	.28	.31	3.47	49	.005**
	Ctrl	.29	.09				
3) REA	Exp	.59	.30	.27	3.43	49	.017*
	Ctrl	.32	.15				
*CK*	Exp	.34	.28	-.06	-.65	49	.528
	Crl	.40	.15				
4) CON	Exp	.39	.28	-.06	-.62	50	.540
	Ctrl	.45	.20				
5) COR	Exp	.30	.29	-.06	-.63	49	.539
	Ctrl	.36	.16				
Overall	Exp	.55	.25	.16	2.89	49	.046^*^
	Ctrl	.39	.13				

DAT: data capture; HYP: hypotheses formulation; REA: reasoning; CON: concept identification; COR: concept relationships

RP: reasoning process; CK: construction of knowledge

Exp: experimental group; Ctrl: control group

*p <0.05; **p <0.01

### Subject-matter knowledge tests

The independent t-tests showed no significant difference between the two groups in the pretest scores (experimental group: *M* = 0.51, *SD* = 0.12; control group: *M* = 0.52, *SD* = 0.19) and posttest scores (experimental group: *M* = 0.56, *SD* = 0.21; control group: *M* = 0.55, *SD* = 0.21).

### Learner perceptions and comments

Learners in both groups reported their perceived learning gains to be moderate in both the reasoning process (experimental group: *M* = 2.17, *SD* = 0.97; control group: *M* = 2.11, *SD* = 0.90) and the construction of knowledge underlying the reasoning process (experimental group: *M* = 2.25, *SD* = 0.89; control group: *M* = 2.26, *SD* = 0.88). No significant differences were found between the two groups.

Responses to the open-ended questions showed that learners in both groups found the learning program to be attractive and stimulating, cultivating their thinking and reasoning skills for self-directed learning with authentic problems. Learners in the experimental group stated that the cognitive-mapping approach was engaging in that it offered a vivid picture of clinical reasoning with connections to interrelated knowledge, although they mentioned that some operations in the cognitive-mapping tool could be simplified. Learners in the control group commented that the learning program enabled them to apply the abstract knowledge to clinical reasoning practice, which in turn stimulated them to reflect on their knowledge gaps. In addition, learners in the control group suggested that feedback from experts be provided to them during the task period. Finally, learners in both groups mentioned that more cases could be offered to allow them to learn more from practical experience.

## Discussion

This study examined the effects of a computer-based cognitive-mapping approach, which helped learners to make sense of the complex reasoning process and the knowledge underlying the reasoning process when they worked with clinical cases. A comparison between the cognitive-mapping approach and the verbal-text approach was made by analyzing their effects on the problem-solving performance, subject-matter knowledge, and learner perceptions and comments.

### Problem-solving performance

*First,* analysis of the learning products found that learners in the cognitive-mapping group outperformed those in the verbal-text group in the reasoning process, but not in the construction of knowledge underlying the reasoning process. The differences in the reasoning process were reflected in learners’ performance in data capture, hypotheses formulation, and reasoning with justifications. *Second*, learners in the cognitive-mapping group made significant pre-post improvement in both the reasoning process and the construction of knowledge underlying the reasoning process. *Third*, learners in the verbal-text group made no apparent pre-post improvement in any aspect of the problem-solving performance.

The findings regarding the effects of the cognitive-mapping approach in supporting the reasoning process, particularly in capturing critical data, formulating hypotheses, and reasoning with justifications, provide empirical evidence of the claimed advantages of graphics-based cognitive-mapping approaches in representing and manipulating cognition in complex problem situations [25]. On the other hand, no significant differences were found between the two approaches regarding their effects on the construction of knowledge underlying the reasoning process, i.e., identifying relevant knowledge and organizing it into a systematic structure. Further studies are needed to examine this issue. Meanwhile, some prior studies noted that concept mapping tasks may place high cognitive demands on learners’ ability to integrate multiple forms of thinking into a complex weave of interrelated concepts [26]. There is a need for more effort to determine effective strategies that guide students’ concept mapping towards meaningful understanding and systematic organization of subject-matter knowledge.

### Knowledge posttest

No significant differences in the posttest scores were found between the two groups, and no apparent pre-post improvement made by both groups. This result is in line with the finding of no significant differences between the two approaches with regard to their effects on the construction of knowledge underlying the reasoning process. Meanwhile, prior studies noted that learning outcomes in problem-solving contexts are mixed and not always fully reflected in traditional tests, and that traditional examinations lack sensitivity to learning in problem-solving contexts in their assessment criteria [20, 27]. Further studies are needed to examine the effects of the cognitive-mapping approach on improving learners’ subject-matter knowledge via problem-solving tasks.

### Learner perceptions and comments

Learners in both groups commented that the learning program was attractive and stimulating, especially in linking clinical practice with subject-matter knowledge; they also requested that more cases be provided in the program. Both groups reported their perceived learning gains to be moderate with regard to the reasoning process and the construction of knowledge underlying the reasoning process, with no significant differences between the two groups. Further, learners in the cognitive-mapping group reported being engaged in the visual-mapping activities, and they requested simplified operations for the cognitive-mapping tool. Learners in the verbal-text group claimed that the learning program had stimulated them to reflect on their knowledge gaps throughout the tasks, and they requested the provision of expert feedback to their task performance.

## Conclusions

It is important to help students to externalize and manage complex, implicit processes involved in learning by working with real-world problems or authentic tasks. Compared with verbal text, visual representations have advantages in representing complex ideas. The findings of this study have shown a promising advantage of the cognitive mapping approach over verbal text in helping learners to externalize and improve problem-solving and reasoning processes. In particular, the cognitive mapping approach extends traditional concept mapping by guiding learners’ attention to a set of key elements of cognition involved in clinical reasoning and in construction of knowledge underling the reasoning process. Meanwhile, cognitive mapping may place a high demand on learners’ capability to integrate multiple forms of thinking into a complex weave of interrelated concepts. Further studies are needed to examine effective strategies to guide students’ cognitive mapping, especially in revealing the knowledge underlying the reasoning process. Such strategies may influence the effects of the cognitive mapping approach in helping learners to construct subject-matter knowledge on the basis of practical experience.

This study was limited in several respects. *First*, volunteer participants may not be representative of the target population, and findings from a small number of participants might restrict their generalization. *Second,* with the lack of random assignment of participants to treatment groups, the results of the study may not convincingly demonstrate a causal link between the treatment condition and observed outcomes. *Third*, the assessment of learning performance based on different types of learning products (cognitive maps from the experimental group, and verbal text from the control group) may affect the results. Fourth, findings from a single domain (kidney disease) might restrict their generalization. Some domains might inherently be more amenable to the cognitive mapping approach than others. These limitations will be taken into account in further studies.
